# Explainable machine learning models for predicting 30-day readmission in pediatric pulmonary hypertension: A multicenter, retrospective study

**DOI:** 10.3389/fcvm.2022.919224

**Published:** 2022-07-26

**Authors:** Minjie Duan, Tingting Shu, Binyi Zhao, Tianyu Xiang, Jinkui Wang, Haodong Huang, Yang Zhang, Peilin Xiao, Bei Zhou, Zulong Xie, Xiaozhu Liu

**Affiliations:** ^1^College of Medical Informatics, Chongqing Medical University, Chongqing, China; ^2^Medical Data Science Academy, Chongqing Medical University, Chongqing, China; ^3^Department of Cardiology, The First Affiliated Hospital of Chongqing Medical University, Chongqing, China; ^4^Department of Cardiology, The Second Affiliated Hospital of Chongqing Medical University, Chongqing, China; ^5^Information Center, The University-Town Hospital of Chongqing Medical University, Chongqing, China; ^6^Department of Urology, Children's Hospital of Chongqing Medical University, Chongqing, China; ^7^Personnel Department, Chongqing Health Center for Women and Children, Chongqing, China

**Keywords:** pediatric pulmonary hypertension, readmission, machine learning, prediction, risk factors

## Abstract

**Background:**

Short-term readmission for pediatric pulmonary hypertension (PH) is associated with a substantial social and personal burden. However, tools to predict individualized readmission risk are lacking. This study aimed to develop machine learning models to predict 30-day unplanned readmission in children with PH.

**Methods:**

This study collected data on pediatric inpatients with PH from the Chongqing Medical University Medical Data Platform from January 2012 to January 2019. Key clinical variables were selected by the least absolute shrinkage and the selection operator. Prediction models were selected from 15 machine learning algorithms with excellent performance, which was evaluated by area under the operating characteristic curve (AUC). The outcome of the predictive model was interpreted by SHapley Additive exPlanations (SHAP).

**Results:**

A total of 5,913 pediatric patients with PH were included in the final cohort. The CatBoost model was selected as the predictive model with the greatest AUC for 0.81 (95% *CI*: 0.77–0.86), high accuracy for 0.74 (95% *CI*: 0.72–0.76), sensitivity 0.78 (95% *CI*: 0.69–0.87), and specificity 0.74 (95% *CI*: 0.72–0.76). Age, length of stay (LOS), congenital heart surgery, and nonmedical order discharge showed the greatest impact on 30-day readmission in pediatric PH, according to SHAP results.

**Conclusions:**

This study developed a CatBoost model to predict the risk of unplanned 30-day readmission in pediatric patients with PH, which showed more significant performance compared with traditional logistic regression. We found that age, LOS, congenital heart surgery, and nonmedical order discharge were important factors for 30-day readmission in pediatric PH.

## Introduction

Pediatric pulmonary hypertension (PH) is a severe and fatal disease characterized by pulmonary vascular remodeling, which increases pulmonary arterial pressure, and is often associated with high mortality, causing a substantial burden on individuals and society (1–3). Unplanned 30-day readmission rates have become a safety and medico-economic issue as a parameter of healthcare quality and a link to a substantial burden on healthcare resources (4–6). A previous study reported a high incidence of 30-day readmission in pediatric PH for 26.3%, leading to a marked increase in associated hospital charges (7). To improve healthcare and relieve the medical burden for pediatric patients with PH, it is necessary to assess the 30-day readmission rates in pediatric PH.

There have been some reports of this problem. Some researchers have proposed that the 30-day readmission rate for pediatric PH may be related to several risk factors, such as age, female gender, congenital heart disease, public insurance, use of inhaled nitric oxide, invasive mechanical ventilation, and the number of PH admissions (7, 8). However, there are currently no risk stratification models to comprehensively assess risk factors with large sample sizes of clinical parameters. Machine learning (ML) is a technique focused on how computers discover underlying patterns from high-dimensional and large datasets, which can be applied in clinical practice to develop efficient and robust predictive models (9, 10). Many studies have shown that models based on ML have better performance than traditional statistical models using the Logistic Regression algorithm (11, 12).

In this study, we aimed to develop prediction models and evaluate the risk factors and causes associated with readmission within 30 days for pediatric PH using ML algorithms. This will allow us to clinically target pediatric patients with PH to reduce readmission rates and improve healthcare quality.

## Materials and methods

### Study population and data source

We retrospectively collected de-identified electronic health record (EHR) data from inpatients at the Chongqing Medical University Medical Data Platform from seven hospitals in Chongqing, China. Pediatric patients (<18 years old) diagnosed with PH according to the International Statistical Classification of Diseases and Related Health Problems, 10th versions (ICD-10) code corresponding to a diagnosis of PH (I27.0, I27.2, P29.3), and discharged between 1 January 2012 and 1 January 2019, were included in this study cohort. The diagnosis of PH was based on clinical findings with echocardiographic confirmation. The first admission and readmission were included when a patient had multiple admissions. Patients who died during admission or were transferred to another hospital or whose discharge status was uncertain were excluded from this study. The present study was approved by the Ethics Committee of Chongqing Medical University. Due to the retrospective observational design, the requirement for informed consent was eliminated. The primary outcome of this study was all-cause unplanned 30-day readmission. The study was reported in accordance with the recommendations of the Transparent Reporting of a multivariable prediction model for Individual Prognosis Or Diagnosis (TRIPOD) statement (13, 14).

### Data collection, data preprocessing and feature selection

A total of 32 variables associated with readmission were collected according to relevant studies and clinical availability (7, 15), including patient demographics (gender and age), length of stay (LOS), etiology of PH, comorbidities, targeted pharmacotherapy for PH during hospitalization, use of mechanical ventilation, and nonmedical order discharge. All data for included variables were extracted from inpatient electronic medical records.

In this study, binary categorical features were encoded as 0 and 1. For instance, the gender of patients was encoded as 0 or 1 (0 = female, 1 = male). Features related to clinical conditions, such as comorbidities, procedures, and medications, were encoded as 0 or 1 (0 = absence, 1 = presence). The primary outcome variable was encoded as 0 or 1 (1 = readmission, 0 = non-readmission).

The least absolute shrinkage and selection operator (LASSO) was applied to identify impactful clinical variables to remove irrelevant and redundant information and improve the discriminative power of ML models (16). The LASSO allows computationally efficient feature selection based on the assumption of linear dependency between input variables and output values and output the regression coefficients of each input variable (17). The variables with non-zero coefficients were selected to construct prediction models in this study.

The entire research process was completed independently by two researchers (MJ Duan and XZ Liu), the final results were checked, and disagreements were discussed with a third researcher (TT Shu) to reach a consensus.

### Model development and performance evaluation

This study randomly divided the dataset into a training set (with 70% objects) and a validation set (with 30% objects) by stratified random sampling. The former was used to develop ML models and the latter was used to evaluate the prediction performance of models.

This study compared the performance of 15 ML algorithms without hyper-parameters optimization to screen the candidate algorithms for 30-day readmission prediction models. The top four algorithms with excellent performance would be selected according to the accuracy and the area under the receiver operating characteristic (ROC) curve (AUC). In this study, Logistic Regression (LR) was selected as a comparison to the traditional statistical model. The screening process was conducted using the PyCaret package (version 2.3.3), an open-source and low-code ML library in Python.

This study constructed five predictive models based on the training set and tuned models with the Bayesian optimization algorithm to select the optimal hyper-parameter configuration. The Bayesian optimization algorithm, an efficient constrained global optimization tool, was performed with the functions of the Bayes_opt Python package (version 1.2.0) and the 10-fold cross-validation method (18–20). During cross-validation, the training set was split into 10 sets, and nine of them were used for model training and one for model evaluation. This study repeated the process 10 times to examine all potential selections of training-evaluation sets.

This study compared the discriminant power among different models calculated in an independent validation set through AUC. Accuracy, sensitivity, and specificity were also calculated. SHapley Additive exPlanations (SHAP) was applied through a SHAP python package (version 0.39.0) to interpret the models to mitigate the black-box nature of ML and help clinicians understand the results provided by models (21). The impact of each input variable on the model output was assessed by Shapley values calculated from coalitional game theory (21, 22).

### Statistical analyses

This study compared the baseline characteristics of pediatric patients with PH between the readmission group and the non-readmission group. Categorical variables were expressed as frequency or proportions and compared by the chi-square test or Fisher's exact test. Continuous variables were shown as mean ± standard deviation (SD) and median with 95% confidence interval (*CI*), also with the first quartile (Q1) and the third quartile (Q3). Continuous variables with normal distribution were analyzed by Student's *t*-test and continuous variables with skewed distribution by the Mann–Whitney *U*-test. A two-sided *p* < 0.05 was considered to be statistically significant. Statistical analyses were performed with an open-source Scipy python package (version 1.7.1) (23).

## Results

### Characteristics of study population

**Figure 1:**
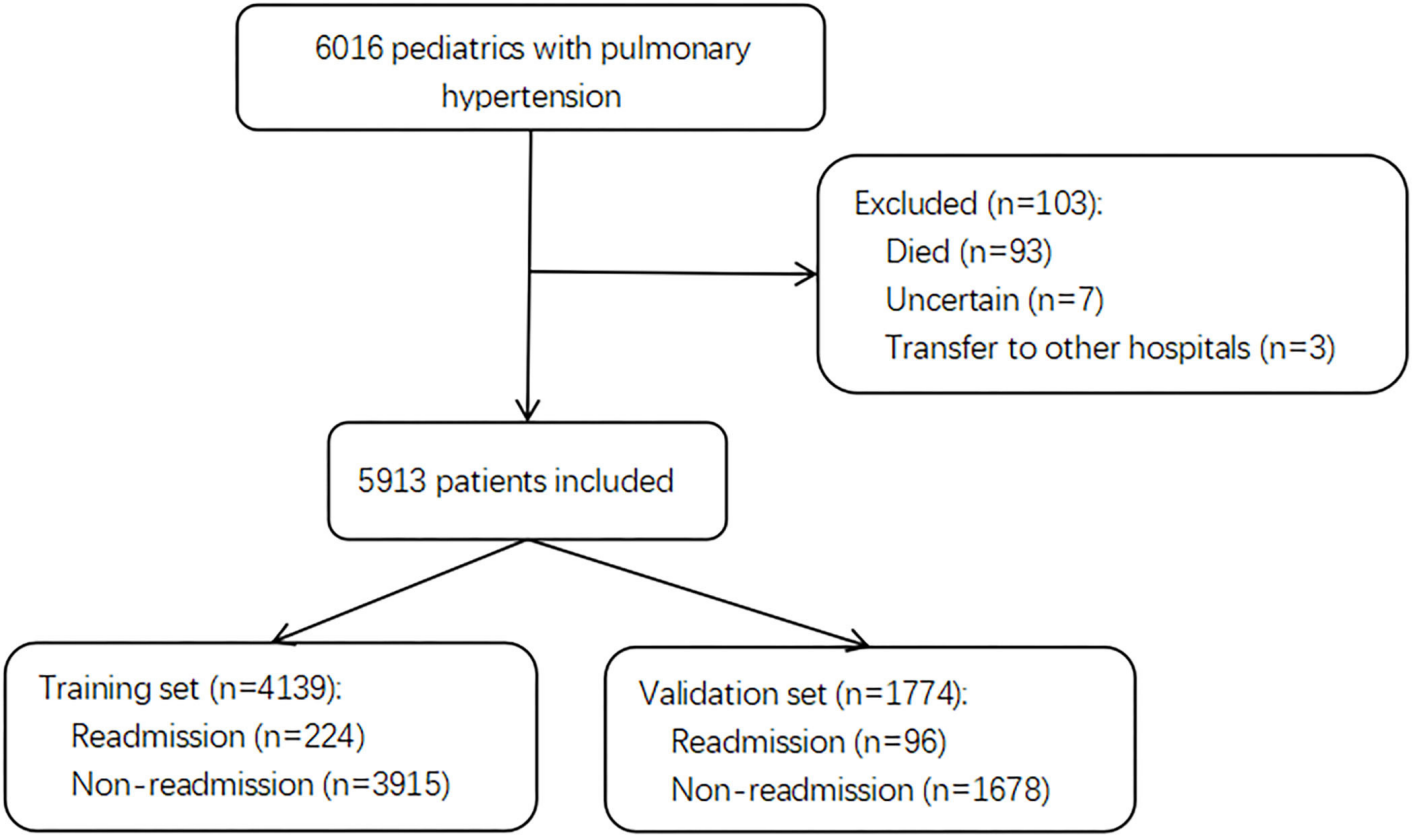
The flowchart of the patient selection.

Of 6,016 pediatric inpatients diagnosed with PH, 103 patients were excluded due to death, transfer to other hospitals, and uncertain discharge during initial admission. A total of 5,913 pediatric patients with PH were included in the final cohort and divided into a training set with 4,139 patients for model building and a validation set with 1,774 patients for testing. The selection flow chart is shown in Figure 1. In total, there were 320 (5.4%) pediatric patients with PH re-admitted within 30 days after index discharge and 5,780 (98%) patients associated with the etiology of congenital heart disease (CHD). The median age was 0.11 years (0.00, 0.60) in total patients, with a proportion of boys of 3,255 (55%). The differences in characteristics between readmission and non-readmission groups are described in Table 1. In the training set and test set, 224 (5.4%) and 96 (5.4%) patients were readmitted within 30 days in this study individually. The baseline characteristics of patients in the two sets are presented in Supplementary Tables 1 and 2, respectively. The baseline characteristics between the training set and validation set are shown in Supplementary Table 3.

**Table 1:** Baseline characteristics of pediatrics with pulmonary hypertension (PH).

**Variables**	**Readmission (*****n*** = **320)**	**Non-readmission** **(*****n*** = **5,593)**	***P*** **-value**
Age (years), median [Q1, Q3]	0.27 [0.09, 0.62]	0.10 [0.00, 0.60]	< 0.001
Male, *n* (%)	185 (57.8)	3,070 (54.9)	0.307
IPAH, *n* (%)	1 (0.3)	56 (1)	0.220
Connective tissue disease, *n* (%)	0 (0)	5 (0.1)	0.593
Dilated cardiomyopathy, *n* (%)	0 (0)	9 (0.2)	0.473
CHD, *n* (%)	316 (98.8)	5,464 (97.7)	0.215
BPD, *n* (%)	12 (3.8)	125 (2.2)	0.080
Interstitial lung disease, *n* (%)	3 (0.9)	31 (0.6)	0.378
Obstructive sleep apneas, *n* (%)	0 (0)	5 (0.1)	0.593
Asthma, *n* (%)	5 (1.6)	30 (0.5)	0.020
Hypothyroidism, *n* (%)	1 (0.3)	24 (0.4)	0.755
Persistent PH in newborn, *n* (%)	2 (0.6)	50 (0.9)	0.616
Congenital diaphragmatic hernia, *n* (%)	1 (0.3)	27 (0.5)	0.666
Chromosomal abnormalities, *n* (%)	13 (4.1)	336 (6.0)	0.151
Preterm birth, *n* (%)	24 (7.5)	912 (16.3)	< 0.001
Low-weight-birth infants, *n* (%)	14 (4.4)	459 (8.2)	0.014
Very-low-birth-weight infants, *n* (%)	3 (0.9)	96 (1.7)	0.291
Sepsis, *n* (%)	26 (8.1)	1,035 (18.5)	< 0.001
Intracranial hemorrhage, *n* (%)	27 (8.4)	1,341 (24.0)	< 0.001
Arrhythmia, *n* (%)	1 (0.3)	67 (1.2)	0.149
Multi-organ dysfunction syndromes, *n* (%)	0 (0)	7 (0.1)	0.527
Respiratory failure, *n* (%)	99 (30.9)	2,042 (36.5)	0.044
Heart failure, *n* (%)	13 (4.1)	178 (3.2)	0.387
Severe pneumonia, *n* (%)	81 (25.3)	845 (15.1)	< 0.001
**Targeted pharmacotherapy**			
Prostacyclin, *n* (%)	3 (0.9)	90 (1.6)	0.348
PDE-5i, *n* (%)	21 (6.6)	716 (12.8)	0.001
Endothelin receptor antagonists, *n* (%)	4 (1.3)	31 (0.6)	0.115
Combination therapy, *n* (%)	0 (0)	3 (0.1)	0.679
Congenital heart surgery, *n* (%)	8 (2.5)	689 (12.3)	< 0.001
Mechanical ventilation, *n* (%)	19 (5.9)	1,509 (27.0)	< 0.001
Nonmedical order discharge, *n* (%)	71 (22.2)	1,805 (32.3)	< 0.001
LOS (days), median [Q1, Q3]	10 [7, 13]	12 [7, 21]	< 0.001

n, number; Q1, the first quartile; Q3, the third quartile; IPAH, idiopathic pulmonary arterial hypertension; CHD, congenital heart disease; BPD, bronchopulmonary dysplasia; PDE-5i, phosphodiesterase 5 inhibitors; LOS, length of stay.

### Candidate algorithms screening

The results of 15 algorithms are exhibited in Table 2. In this study, accuracy and AUC were defined as the main parameters to evaluate the models' performance. The top four algorithms with excellent performance (accuracy > 0.9 and AUC > 0.7) were selected to develop the readmission prediction models, such as eXtreme Gradient Boosting (XGBoost), Random Forest, Categorical Boosting (CatBoost), and Light Gradient Boosting Machine (LightGBM). Logistic Regression (LR), a traditional statistical model, was selected as a comparison in this study.

**Table 2:** Performance of different models.

	**Model**	**Accuracy**	**AUC**
1	eXtreme Gradient Boosting	0.9063	0.7474
2	Random Forest Classifier	0.9050	0.7284
3	Light Gradient Boosting Machine	0.9021	0.7571
4	CatBoost Classifier	0.9000	0.7521
5	Extra Trees Classifier	0.8973	0.6879
6	Decision Tree Classifier	0.8761	0.5802
7	Gradient Boosting Classifier	0.8524	0.7640
8	K Neighbors Classifier	0.8154	0.6455
9	Ada Boost Classifier	0.8084	0.7584
10	SVM-Linear Kernel	0.7047	0.0000
11	Logistic Regression	0.5963	0.7085
12	Ridge Classifier	0.5825	0.0000
013	Linear Discriminant Analysis	0.5820	0.7059
14	Naive Bayes	0.1819	0.5829
15	Quadratic Discriminant Analysis	0.0916	0.5072

AUC, area under the curve.

### Development of prediction models

**Figure 2:**
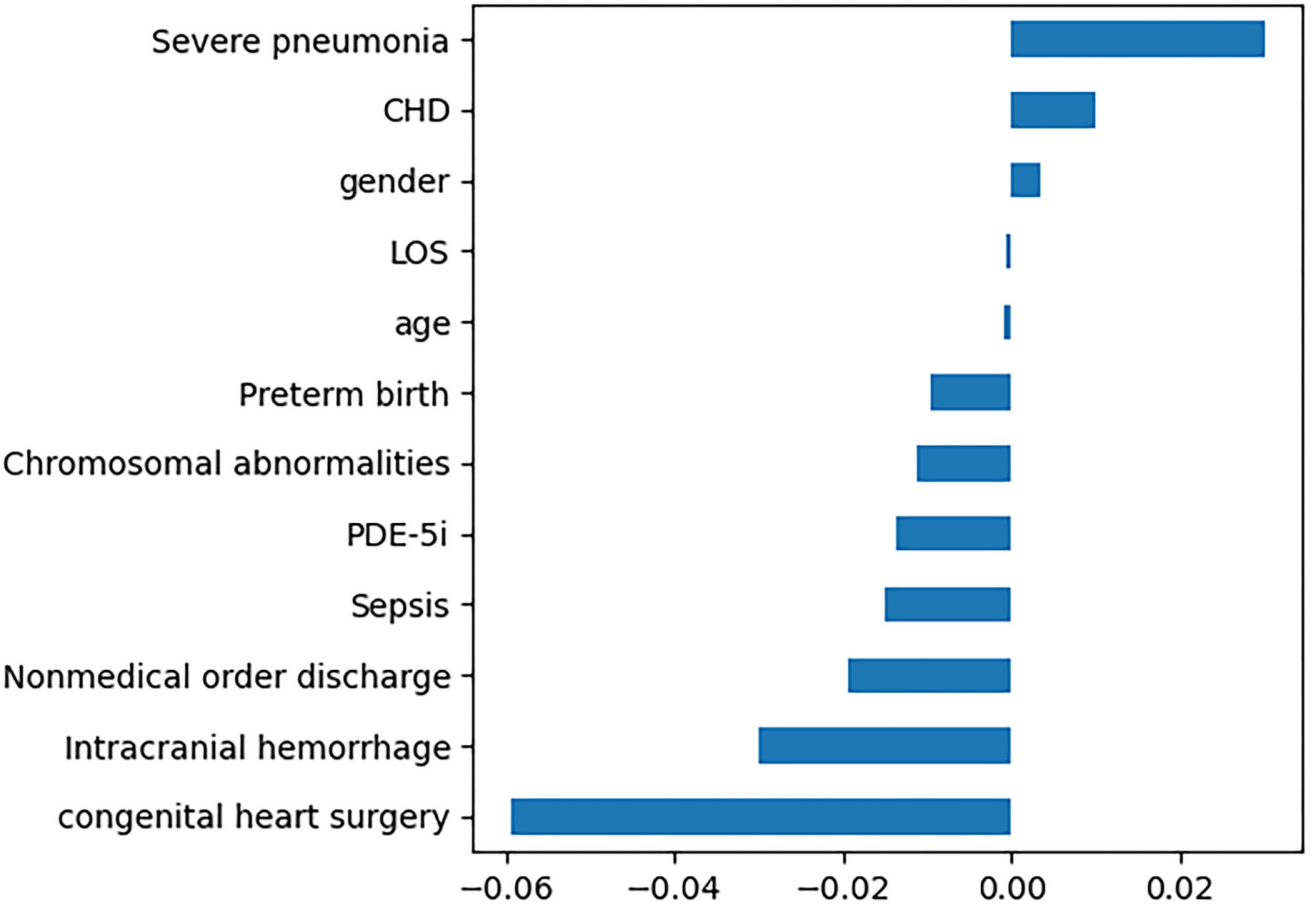
Twelve features with non-zero regression coefficients. LOS, length of stay; PDE-5i, phosphodiesterase 5 inhibitors; CHD, congenital heart disease.

In this study, 12 features with non-zero regression coefficients were selected to construct predictive models through LASSO feature selection analysis shown in Figure 2, such as severe pneumonia, CHD, gender, LOS, age, preterm birth, chromosomal abnormalities, phosphodiesterase 5 inhibitors (PDE-5i), sepsis, nonmedical order discharge, intracranial hemorrhage, and congenital heart surgery. Bayesian optimization was conducted to optimize the hyperparameters.

**Figure 3:**
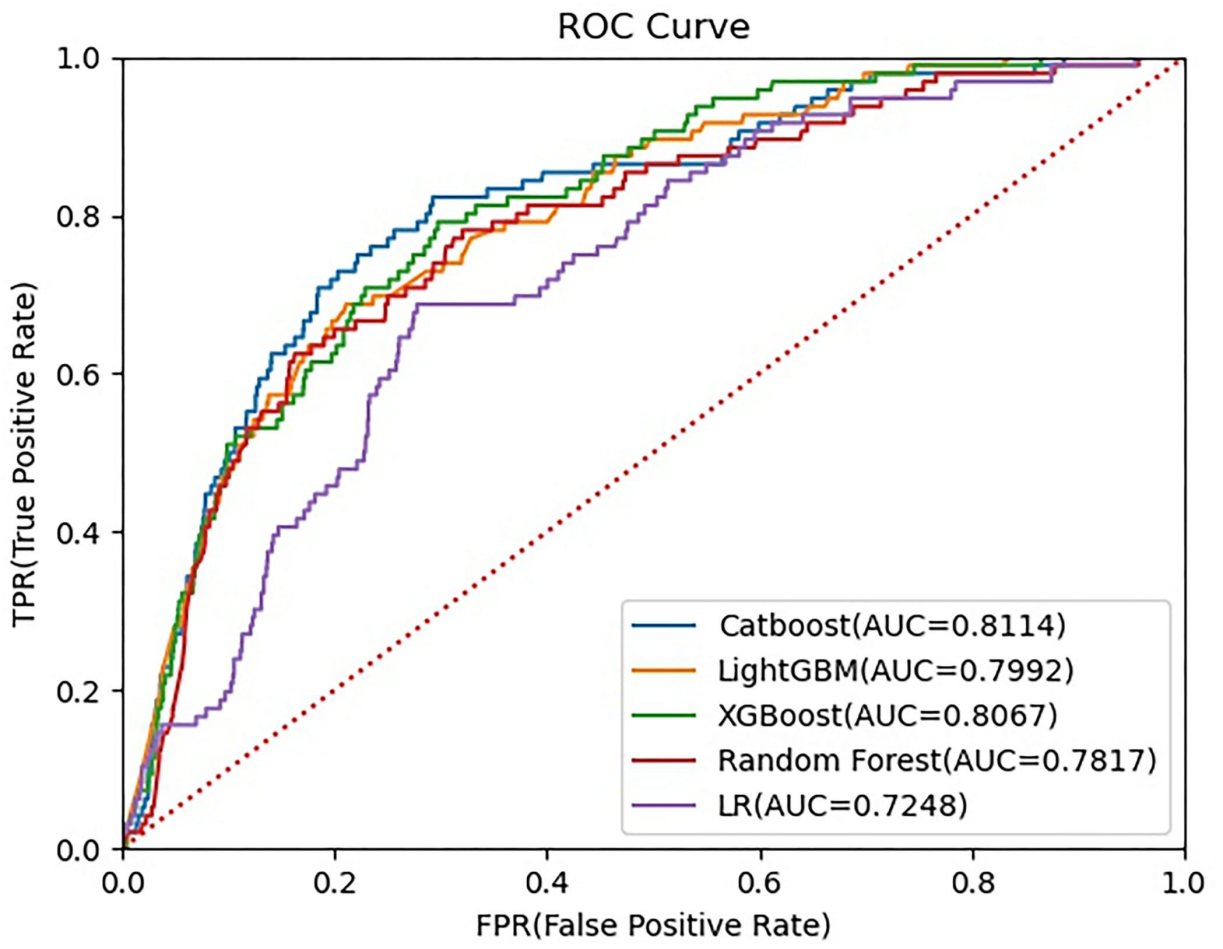
Receiver operating characteristic (ROC) curve for five machine learning-based prediction models. ROC, receiver operating characteristic curve; LightGBM, Light Gradient Boosting Machine; XGBoost, eXtreme gradient boosting; LR, logistic regression.

The ROC curves of five constructed models are shown in Figure 3. The CatBoost model showed the greatest AUC with 0.8114 (95% *CI*: 0.7662–0.8551). The AUC of XGBoost model was 0.8067 (95% *CI*: 0.7657–0.8465), the LightGBM model 0.7992 (95% *CI*: 0.7560–0.8407), the Random Forest model 0.7817 (95% *CI*: 0.7335–0.8277), and the LR model 0.7248 (95% *CI*: 0.6765–0.7723), respectively. This study calculated the accuracy, sensitivity, and specificity of the selected models to evaluate the performance of the models comprehensively, as shown in Table 3. The CatBoost model was finally selected as the predictive model for unplanned 30-day readmission in pediatric PH, with a high accuracy for 0.7401 (95% *CI*: 0.7198–0.7604), sensitivity 0.7813 (95% *CI*: 0.6907–0.8660), and specificity 0.7378 (95% *CI*: 0.7167–0.7585). The hyperparameter search domain and final setting of the CatBoost model are listed in Supplementary Table 4. The predictive performance of the CatBoost model for different gender and age sub-cohorts in the independent validation set is shown in Table 4.

**Table 3:** Performance evaluation of the 5 prediction models.

**Models**	**AUC** **(95% CI)**	**Accuracy** **(95% CI)**	**Sensitivity (95% CI)**	**Specificity** **(95% CI)**
CatBoost	0.8114 (0.7662–0.8551)	0.7401 (0.7198–0.7604)	0.7813 (0.6907–0.8660)	0.7378 (0.7167–0.7585)
XGBoost	0.8067 (0.7657–0.8465)	0.7458 (0.7255–0.7655)	0.7188 (0.6180–0.8085)	0.7473 (0.7258–0.7677)
LightGBM	0.7992 (0.7560–0.8407)	0.6855 (0.6629–0.7069)	0.7396 (0.6500–0.8256)	0.6824 (0.6597–0.7040)
Random Forest	0.7817 (0.7335–0.8277)	0.7012 (0.6798–0.7227)	0.7396 (0.6489–0.8246)	0.6990 (0.6772–0.7205)
Logistic Regression	0.7248 (0.6765–0.7723)	0.5186 (0.4943–0.5428)	0.8125 (0.7304–0.8889)	0.5018 (0.4767–0.5262)

AUC, area under the curve; CI, confidence interval; LightGBM, light gradient boosting machine; XGBoost, eXtreme gradient boosting.

**Table 4:** Performance evaluation of the CatBoost model using the validation subset.

	**N**	**AUC** **(95% CI)**	**Accuracy (95% CI)**	**Sensitivity** **(95% CI)**	**Specificity (95% CI)**
Male	945	0.8192 (0.7689–0.8684)	0.7566 (0.7291–0.7841)	0.7857 (0.6786–0.8873)	0.7548 (0.7260–0.7843)
Female	829	0.8015 (0.7228–0.8750)	0.7214 (0.6900–0.7515)	0.7750 (0.6389–0.9000)	0.7186 (0.6866–0.7487)
**Age**					
<1 years	1464	0.8102 (0.7612–0.8570)	0.7575 (0.7363–0.7794)	0.7901 (0.6974–0.8765)	0.7556 (0.7332–0.7780)
1–6 years	247	0.7963 (0.6887–0.8902)	0.6559 (0.5951–0.7126)	0.7143 (0.4545–0.9333)	0.6524 (0.5880–0.7105)
6–18 years	63	0.9839 (NA)	0.6667 (0.5556–0.7778)	1.000 (NA)	0.6613 (0.5484–0.7778)

N, number; AUC, area under the curve; CI, confidence interval; NA, not applicable.

### Model interpretation

**Figure 4:**
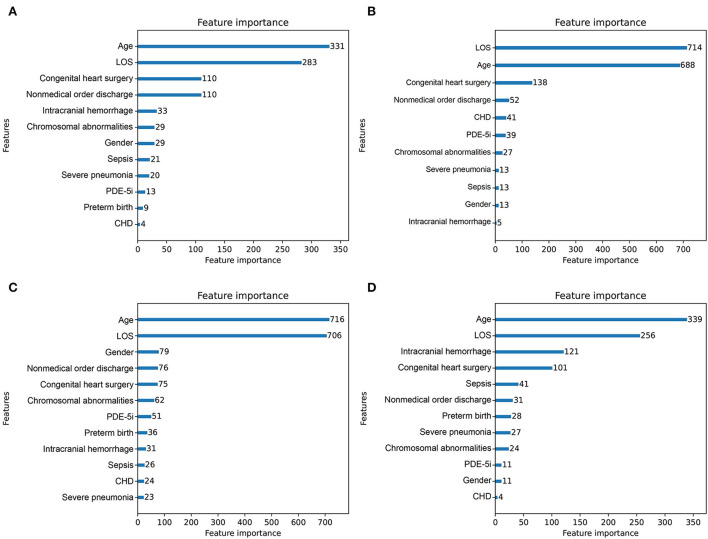
Importance score ranking of features in 4 readmission-predicting algorithms. **(A)** CatBoost. **(B)** Light Gradient Boosting Machine. **(C)** eXtreme gradient boosting. **(D)** Random forest.

Feature importance rankings of four prediction models are shown in Figure 4, including CatBoost (A), LightGBM (B), XGBoost (C), and Random forest (D). The importance scores were calculated by the built-in attributes in different ML algorithms. The most related factors for unplanned 30-day readmission in pediatric patients with PH were age, LOS, congenital heart surgery, and nonmedical order discharge, with slight differences in importance ranking.

**Figure 5:**
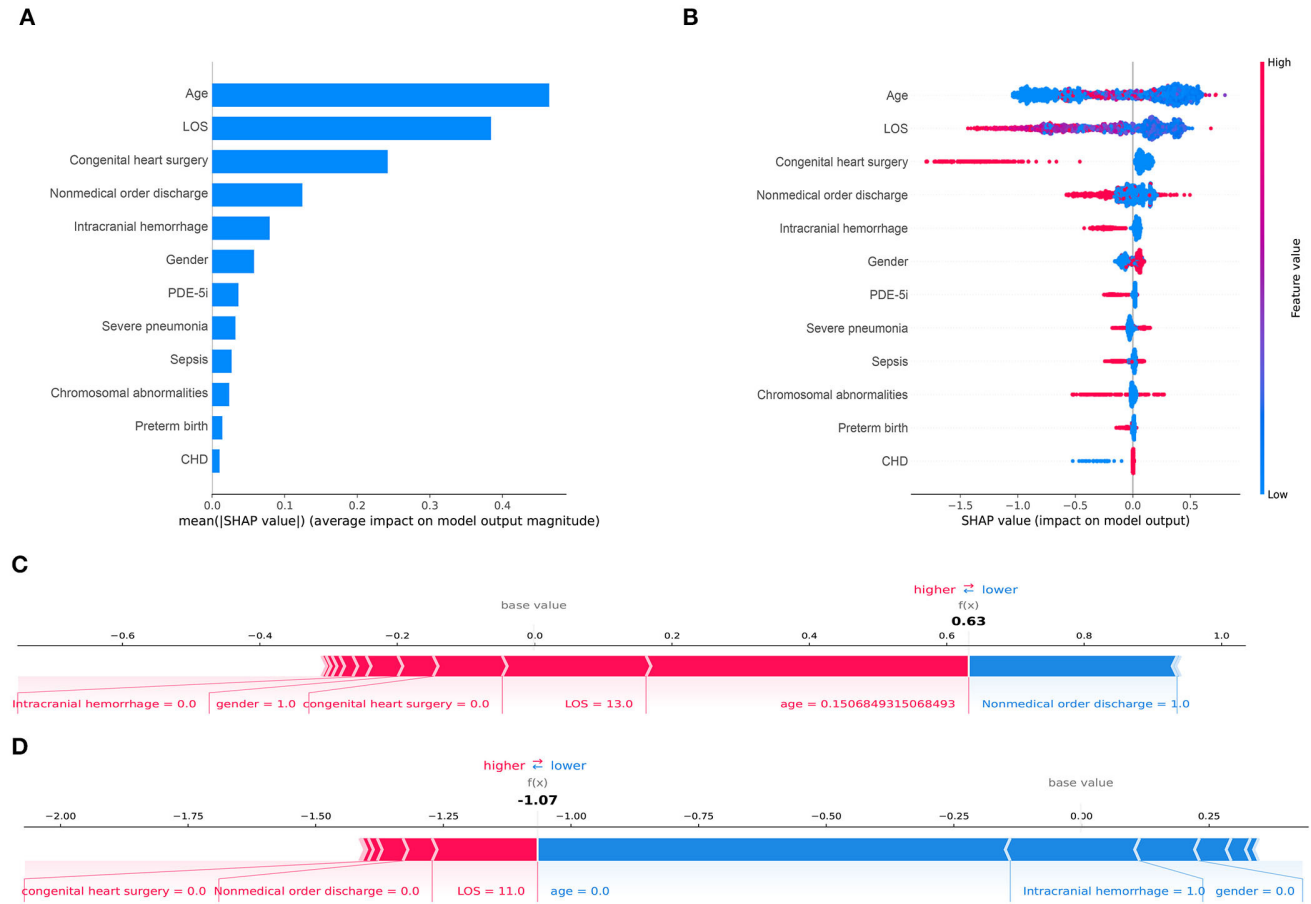
Shapley Additive Explanations (SHAP) for the CatBoost model. **(A)** shows the most impactful features on prediction (ranked from most to least important). **(B)** shows the distribution of the impacts of each feature on the model output. Within each row, each dot represents a patient. The colors of the dots represent the feature values: red for larger values and blue for lower. **(C, D)** show the individualized predictions for two patients. The bars in red and blue represent risk factors and protective factors, respectively; longer bars represent greater feature importance. LOS, length of stay; PDE-5i, phosphodiesterase 5 inhibitors; CHD, congenital heart disease.

This study analyzed the independent validation set in the CatBoost model through the Tree-Explainer class imported from the SHAP package, shown in Figure 5. In the final selected predictive model, the CatBoost model, the related features ranked with the importance score according to the SHAP summary plots to unplanned 30-day readmission in pediatric PH were age, LOS, congenital heart surgery, nonmedical order discharge, intracranial hemorrhage, gender, PDE-5i, severe pneumonia, sepsis, chromosomal abnormalities, preterm birth, and CHD (Figures 5A,B). The results of the independent validation set were consistent with the results shown in Figure 4A.

This study displayed two individual samples with prediction and explanation computed by the force plot method of the SHAP package in Figures 5C,D. The PH patient in Figure 5C was a 55-day old boy with no congenital heart surgery and no intracranial hemorrhage, who had LOS for 13 days and was discharged without a medical order. In this model, the predicted probability for unplanned 30-day readmission of this patient was 63% compared with the base value of about −1%. The PH patient in Figure 5D was a newborn girl with intracranial hemorrhage and no congenital heart surgery, who had LOS for 11 days and was discharged with a medical order. The predicted probability for unplanned 30-day readmission of this patient by the model was −107% compared with the baseline of about −1%. In the predictive models, all listed features shown by the red bars increased the risk of unplanned 30-day readmission in pediatric patients with PH, whereas listed features with blue bars decreased the risk.

## Discussion

Previous studies have shown that unplanned readmission of pediatric PH within a short term is potentially preventable (24). Preventable readmission results in unnecessary hospitalization, an increased risk of nosocomial infections, additional healthcare expenditures, the waste of medical resources, and an increased burden on society and individuals (25). Therefore, it is vital and promising to construct a prediction model with unplanned 30-day readmission in pediatric PH for clinicians. It may produce a positive effect on medicine and finance.

The present study was the first research to develop a risk stratification model with ML for predicting and explaining the unplanned 30-day readmission in pediatric patients with PH based on the EHRs data collected from a database in Chongqing, China. From the five ML models developed and validated based on 12 relevant clinical variables, the CatBoost model showed the best performance and identified factors strongly associated with unplanned 30-day readmission, such as age, LOS, congenital heart surgery, and nonmedical order discharge in pediatric patients with PH.

The prominent etiological type in pediatric patients with PH is CHD, which causes clinical presentation, and outcomes in children differ from those in adults (26). A previous study has reported a high unplanned 30-day readmission rate of 26.3% in pediatric PH (7). In this study, we found the incidence of readmission within 30 days in pediatric PH was 5.4%, which was quite lower than the previous investigation. One reason may be that a part of the patient population included in this study were children under 1 year in which patients with transient PH would turn to normal pulmonary arterial pressure with physical development (27). On the other hand, the clinical outcomes of pediatric patients with PH have been improved significantly due to the advancement of therapeutic medications and strategies (26). In addition, the EHRs data collected from a limited database might cause a selection bias and the 30-day readmission rate in pediatric patients with PH requires evidence from multi-center and big data analyses in the future.

With advanced medical informatics, ML has become a promising tool for predicting the outcomes of patients. However, the nature of the ML, black box, hinders its application in clinical practice (28). Transparency and interpretability of ML is vital to make the results understandable (29). The predictive model of this study found several factors that could increase the unplanned 30-day readmission risk of pediatric PH: age in a range, short LOS, no congenital heart surgery, and discharge with medical order, according to SHAP results. Pediatric patients with PH, especially neonates, have a certain proportion of transient PH in which PH will disappear spontaneously with age (27). It is easier for older children with PH to determine treatment options to control clinical symptoms and disease progression because their organ development tends to be stable (30). From the present study, the factor of age is inevitable in clinical. It is effective for pediatric patients with PH to receive regular high-frequency therapy, such as nitric oxide inhalation, and the time required for each hospitalization is short, leading to an increased readmission rate (31). Physicians can reduce the 30-day readmission rate of pediatric PH by developing a long-term stable treatment regimen and following up patients in an outpatient setting. In this study, CHD was the predominant etiology in pediatric PH. This result is consistent with the reported epidemiology (32). For children without cardiac repair surgery, PH could increase the incidence of hypoxemia and heart failure, and the clinical symptoms will be more severe. Pediatric PH is an incurable disease with high mortality. Therefore, early repaired surgery in pediatric patients with CHD may reduce the 30-day readmission rate. Generally, patients discharging with medical orders are told to receive treatment and checkups regularly (30). Some critically sick patients, whose guardians will choose to stop therapy and discharge without a medical order due to medical expenses and other reasons, have an extremely high risk of death after discharge. This study found that the incidence of intracranial hemorrhage could reduce the risk of readmission in pediatric with PH. The median age of pediatrics included in this study was 0.11 years. Previous study have reported an increased incidence of intracranial hemorrhage in term and near-term infants with persistent PH (33, 34). There was a significant association between intracranial hemorrhage and death or neurodevelopmental impairment in infants (35), which could cause the reduction of 30-days readmission in pediatric with PH. With the consideration of identified factors in the present study, physicians need to design a reasonable and individualized discharge plan for pediatric patients.

The present study found that decision tree-based gradient boosting ensemble models showed better performance than the traditional logistic regression. For the validation set, although both the CatBoost model and the XGBoost model showed comparable performance, we choose the CatBoost model as our final prediction model due to the following reasons. One important advantage of CatBoost algorithms is that they handle categorical features automatically (36). Another reason is that the CatBoost model in this study had a higher sensitivity than the XGBoost model, which ensured an extremely low number of false negatives. In recent studies, the CatBoost algorithm was applied to develop prediction models and showed better discrimination capability (37–39). Zhao QY et al. developed a model for predicting extubation failure in intensive care units with 11 ML algorithms and the CatBoost algorithm showed the best performance in the internal and prospective validation set (37). Lo YT et al. built a risk stratification tool for predicting 14-day unplanned readmission, in which the CatBoost algorithm showed the best performance in the 5-fold cross-validation (AUROC:0.9903) of four selected ML algorithms (38).

Subgroup analysis in this study demonstrated that the CatBoost model performance was comparable in male and female patients. The model prediction power measured by AUC in groups of 0–6 years was consistent with the performance in the total validation set. However, the model did not show a reliable predictive performance in groups of 6–18 years, which was indicated by the high AUC and sensitivity with uncalculated 95% *CI*. This can be explained by a low representation of patients in this age range in the total validation set (3.5%).

The present study has several limitations. First, the data used in this study are a small-scale dataset collected from the electronic medical records of local hospitals in Chongqing, China, and each hospital was populated with data to varying degrees. This resulted in significantly missing data for some of the extracted clinical features. Second, for the included pediatrics, the diagnosis of PH was based on echocardiography. That is not the golden and definite diagnosis of PH and may weaken the results. Since we could not derive pictures from the Chongqing Medical University Medical Data Platform, this study lacked some key echocardiographic parameters, such as pulmonary artery systolic pressure. Therefore, these effect indicators were not included in the outcomes of our research. However, the inclusion information was based on discharge diagnosis, so this factor did not affect the overall analysis. Third, the variables included in this study were mainly comorbidities. To ensure the performance of the prediction model and the convenience for clinical application, the objective results, such as vital signs, laboratory tests, and auxiliary examination of patients at admission or discharge were not included as variables for the construction of the model, which may also have important implications for the prognosis of patients. Then, this model was not applied in clinical practice, thus, prospective and external validation was required to further confirm the generalization ability of this model.

## Conclusion

This study developed a CatBoost model to predict the risk of unplanned 30-day readmission in pediatric patients with PH, which showed more significant performance compared with traditional logistic regression. We found that age, LOS, congenital heart surgery, and nonmedical order discharge were important factors for 30-day readmission in pediatric PH. This study has laid a foundation for further research to improve the accuracy of predicting readmission risk.

## Data availability statement

The data analyzed in this study is subject to the following licenses/restrictions: due to ethics committee regulations, the dataset supporting the findings of this study is limited and not public. Requests to access these datasets should be directed to XL, http://www.xiaozhuliu2021@163.com.

## Ethics statement

The data involving human participants in this study were reviewed and approved by the Ethics Committee of Chongqing Medical University. Written informed consent for participation was not required for this study following the national legislation and the institutional requirements.

## Author contributions

XL and ZX is the corresponding author who contributed to the conception of the study. MD and BZ performed the model development and drafted the manuscript. TS contributed to manuscript preparation and constructive discussions. TX, HH, and YZ contributed to the collection of data. PX, BZ, and JW contributed in reviewing the manuscript. All authors have read and approved the final manuscript.

## Funding

This study was supported by the Intelligent Medicine Research Project of Chongqing Medical University (Nos. ZHYX2019013 and YJSZHYX202119) and Chongqing Postdoctoral Program (No. 2010010006118105).

## Conflicts of interest

The authors declare that the research was conducted in the absence of any commercial or financial relationships that could be construed as a potential conflict of interest.

## Publisher's note

All claims expressed in this article are solely those of the authors and do not necessarily represent those of their affiliated organizations, or those of the publisher, the editors and the reviewers. Any product that may be evaluated in this article, or claim that may be made by its manufacturer, is not guaranteed or endorsed by the publisher.

## References

[1] Hopper RK Abman SH Ivy DD. Persistent challenges in pediatric pulmonary hypertension. Chest. (2016) 150:226–36. 10.1016/j.chest.2016.01.007 26836930 PMC6026244

[2] Maxwell BG Nies MK Ajuba-Iwuji CC Coulson JD Romer LH. Trends in hospitalization for pediatric pulmonary hypertension. Pediatrics. (2015) 136:241–50. 10.1542/peds.2014-3834 26148956

[3] Frank DB Crystal MA Morales DLS Gerald K Hanna BD Mallory GB. Trends in pediatric pulmonary hypertension-related hospitalizations in the United States from 2000-2009. Pulm Circ. (2015) 5:339–48. 10.1086/681226 26064460 PMC4449246

[4] Berry JG Toomey SL Zaslavsky AM Jha AK Nakamura MM Klein DJ. Pediatric readmission prevalence and variability across hospitals. JAMA. (2013) 309:372–80. 10.1001/jama.2012.188351 23340639 PMC3640861

[5] Lawson EH Hall BL Louie R Ettner SL Zingmond DS Han L. Association between occurrence of a postoperative complication and readmission implications for quality improvement and cost savings. Ann Surg. (2013) 258:10–8. 10.1097/SLA.0b013e31828e3ac3 23579579

[6] Jukic M Antisic J Pogorelic Z. Incidence and causes of 30-day readmission rate from discharge as an indicator of quality care in pediatric surgery. Acta Chir Belg. 13:1–5. 10.1080/00015458.2021.1927657 33960261

[7] Awerbach JD Mallory GB Kim S Cabrera AG. Hospital readmissions in children with pulmonary hypertension: a multi-institutional analysis. J. Pediatr. (2018) 195:95–101.e4. 10.1016/j.jpeds.2017.11.027 29336798

[8] Sehgal M Amritphale A Vadayla S Mulekar M Batra M Amritphale N. Demographics and risk factors of pediatric pulmonary hypertension readmissions. Cureus. (2021) 13:e18994. 10.7759/cureus.18994 34853737 PMC8608354

[9] Sidey-Gibbons JAM Sidey-Gibbons CJ. Machine learning in medicine: a practical introduction. BMC Med Res Methodol. (2019) 19:64. 10.1186/s12874-019-0681-4 30890124 PMC6425557

[10] Johnson KW Soto JT Glicksberg BS Shameer K Miotto R Ali M. Artificial intelligence in cardiology. J Am Coll Cardiol. (2018) 71:2668–79. 10.1016/j.jacc.2018.03.521 29880128

[11] Feng JZ Wang Y Peng J Sun MW Zeng J Jiang H. Comparison between logistic regression and machine learning algorithms on survival prediction of traumatic brain injuries. J Crit Care. (2019) 54:110–6. 10.1016/j.jcrc.2019.08.010 31408805

[12] Lv HC Yang XL Wang BY Wang SB Du XY Tan Q. Machine learning-driven models to predict prognostic outcomes in patients hospitalized with heart failure using electronic health records: retrospective study. J Med Internet Res. (2021) 23:e24996. 10.2196/24996 33871375 PMC8094022

[13] Collins GS Reitsma JB Altman DG Moons KGM. Transparent reporting of a multivariable prediction model for individual prognosis or diagnosis (TRIPOD): the TRIPOD statement. J Clin Epidemiol. (2015) 68:112–21. 10.1016/j.jclinepi.2014.11.010 25579640

[14] Moons KGM Altman DG Reitsma JB Ioannidis JPA Macaskill P Steyerberg EW. Transparent reporting of a multivariable prediction model for Individual Prognosis Or Diagnosis (TRIPOD): explanation and elaboration. Ann Intern Med. (2015) 162:W1–W73. 10.7326/M14-0698 25560730

[15] Bhattacharya PT Hameed AMA Bhattacharya ST Chirinos JA Hwang WT Birati EY. Risk factors for 30-day readmission in adults hospitalized for pulmonary hypertension. Pulm Circ. (2020) 10:2045894020966889. 10.1177/2045894020966889 33282194 PMC7686634

[16] Chandrashekar G Sahin F. A survey on feature selection methods. Comput Electr Eng. (2014) 40:16–28. 10.1016/j.compeleceng.2013.11.024

[17] Yamada M Jitkrittum W Sigal L Xing EP Sugiyama M. High-dimensional feature selection by feature-wise kernelized Lasso. Neural Comput. (2014) 26:185–207. 10.1162/NECO_a_00537 24102126

[18] Kim Y Chung M. An approach to hyperparameter optimization for the objective function in machine learning. Electronics. (2019) 8:1267. 10.3390/electronics8111267

[19] Joy TT Rana S Gupta S Venkatesh S. Batch Bayesian optimization using multi-scale search. Knowledge-Based Systems. (2020) 187:104818. 10.1016/j.knosys.2019.06.026

[20] Wong TT. Performance evaluation of classification algorithms by k-fold and leave-one-out cross validation. Pattern Recognit. (2015) 48:2839–46. 10.1016/j.patcog.2015.03.009

[21] Lundberg SM Erion G Chen H DeGrave A Prutkin JM Nair B. From local explanations to global understanding with explainable AI for trees. Nat Mach Intell. (2020) 2:56–67. 10.1038/s42256-019-0138-9 32607472 PMC7326367

[22] Nohara Y Matsumoto K Soejima H Nakashima N. Explanation of machine learning models using shapley additive explanation and application for real data in hospital. Computer Methods and Programs in Biomedicine. (2022) 214. 10.1016/j.cmpb.2021.106584 34942412

[23] Virtanen P Gommers R Oliphant TE Haberland M Reddy T Cournapeau D. SciPy 1.0: fundamental algorithms for scientific computing in Python. Nat Methods. (2020) 17:261–72. 10.1038/s41592-019-0686-2 32015543 PMC7056644

[24] Auerbach AD Kripalani S Vasilevskis EE Sehgal N Lindenauer PK Metlay JP. Preventability and causes of readmissions in a national cohort of general medicine patients. JAMA Intern Med. (2016) 176:484–93. 10.1001/jamainternmed.2015.7863 26954564 PMC6900926

[25] Hall BL Namazie-Kummer S. Potentially preventable readmissions after surgery. JAMA Network Open. (2021) 4:e216389. 10.1001/jamanetworkopen.2021.6389 33847755

[26] Rosenzweig EB Abman SH Adatia I Beghetti M Bonnet D Haworth S. Paediatric pulmonary arterial hypertension: updates on definition, classification, diagnostics and management. Eur Respir J. (2019) 53:1801916. 10.1183/13993003.01916-2018 30545978 PMC6351335

[27] van Loon RLE Roofthooft MTR Hillege HL ten Harkel ADJ van Osch-Gevers M Delhaas T. Pediatric Pulmonary Hypertension in the Netherlands Epidemiology and Characterization During the Period. 1991 to 2005. Circulation. (2011) 124:1755–U136. 10.1161/CIRCULATIONAHA.110.969584 21947294

[28] Vellido A. The importance of interpretability and visualization in machine learning for applications in medicine and health care. Neural Comput Appl. (2020) 32:18069–83. 10.1007/s00521-019-04051-w

[29] Miller T. Explanation in artificial intelligence: insights from the social sciences. Artificial Intelligence. (2019) 267:1–38. 10.1016/j.artint.2018.07.007

[30] Hansmann G Koestenberger M Alastalo TP Apitz C Austin ED Bonnet D. Zartner: 2019 updated consensus statement on the diagnosis and treatment of pediatric pulmonary hypertension: the European Pediatric Pulmonary Vascular Disease Network (EPPVDN), endorsed by AEPC, ESPR and ISHLT. J Heart Lung Transplant. (2019) 38:879–901. 10.1016/j.healun.2019.06.022 31495407

[31] Hansmann G. Pulmonary hypertension in infants, children, young adults. J Am Coll Cardiol. (2017) 69:2551–69. 10.1016/j.jacc.2017.03.575 28521893

[32] Mukherjee D Konduri GG. Pediatric pulmonary hypertension: definitions, mechanisms, diagnosis, and treatment. Compr Physiol. (2021) 11:2135–90. 10.1002/cphy.c200023 34190343 PMC8289457

[33] Oelberg DG Temple DM Haskins KS Bigelow RH Adcock EW. Intracranial hemorrhage in term or near-term newborns with persistent pulmonary hypertension. Clin Pediatr. (1988) 27:14–7. 10.1177/000992288802700103 3335111

[34] Gupta SN Kechli AM Kanamalla US. Intracranial hemorrhage in term newborns: management and outcomes. Pediatr Neurol. (2009) 40:1–12. 10.1016/j.pediatrneurol.2008.09.019 19068247

[35] Law JB Wood TR Gogcu S Comstock BA Dighe M Perez K. Intracranial hemorrhage and 2-year neurodevelopmental outcomes in infants born extremely preterm. J Pediatr. (2021) 238:124–134.e10. 10.1016/j.jpeds.2021.06.071 34217769 PMC8551011

[36] Hancock JT Khoshgoftaar TM. CatBoost for big data: an interdisciplinary review. J Big Data. (2020) 7:94. 10.1186/s40537-020-00369-8 33169094 PMC7610170

[37] Zhao QY Wang H Luo JC Luo MH Liu LP Yu SJ. Development and validation of a machine-learning model for prediction of extubation failure in intensive care units. Front Med. (2021) 8:676343. 10.3389/fmed.2021.676343 34079812 PMC8165178

[38] Lo YT Liao JCH Chen MH Chang CM Li CT. Predictive modeling for 14-day unplanned hospital readmission risk by using machine learning algorithms. BMC Medical Informatics and Decision Making. (2021) 21:288. 10.1186/s12911-021-01639-y 34670553 PMC8527795

[39] Zhang CY Chen XF Wang S Hu JJ Wang CP Liu X. Using CatBoost algorithm to identify middle-aged and elderly depression, national health and nutrition examination survey 2011-2018. Psychiatry Res. (2021) 306:114261. 10.1016/j.psychres.2021.114261 34781111

