# Supplementary material for: Explainable machine learning models for predicting 30-day readmission in pediatric pulmonary hypertension: A multicenter, retrospective study
Source: Front Cardiovasc Med. 2022 Jul 26;9:919224. doi: 10.3389/fcvm.2022.919224 (PMC9360407; doi:10.3389/fcvm.2022.919224)
Supplement: Supplementary file 1 [file Data_Sheet_1.pdf]

Supplementary Material

Supplementary Table 1 Baseline characteristic in training set.

Variables	Readmission (n = 224)	Non-readmission (n = 3915)	P-value
Age (years), median [Q1, Q3]	0.25 [0.08, 0.61]	0.09 [0.00, 0.58]	< 0.001
Male, n (%)	129 (57.6)	2181 (55.7)	0.582
IPAH, n (%)	1 (0.4)	32 (0.8)	0.544
Connective tissue disease, n (%)	0 (0)	4 (0.1)	0.632
Dilated cardiomyopathy, n (%)	0 (0)	4 (0.1)	0.632
CHD, n (%)	221 (98.7)	3822 (97.6)	0.316
BPD, n (%)	6 (2.7)	86 (2.2)	0.634
Interstitial lung disease, n (%)	1 (0.4)	23 (0.6)	0.787
Obstructive sleep apneas, n (%)	0 (0)	4 (0.1)	0.632
Asthma, n (%)	2 (0.9)	19 (0.5)	0.404
Hypothyroidism, n (%)	1 (0.4)	18 (0.5)	0.977
Persistent PH in newborn, n (%)	2 (0.9)	39 (1.0)	0.879
Congenital diaphragmatic hernia, n (%)	0 (0.3)	19 (0.5)	0.296
Chromonosomal abnormalities, n (%)	11 (4.9)	236 (6.0)	0.492
Preterm birth, n (%)	18 (8.0)	668 (17.1)	< 0.001
Low-weight-birth infants, n (%)	11 (4.9)	331 (8.5)	0.061

Very-low-birth-weight infants, n (%)	3 (1.3)	68 (1.7)	0.656
Sepsis, n (%)	20 (8.9)	728 (18.6)	<0.001
Intracranial hemorrhage, n (%)	21 (9.4)	966 (24.7)	< 0.001
Arrhythmia, n (%)	1 (0.4)	42 (1.1)	0.369
Multi-organ dysfunction syndromes, n (%)	0 (0)	5 (0.1)	0.593
Respiratory failure, n (%)	77 (34.4)	1426 (36.4)	0.535
Heart failure, n (%)	9 (4.0)	134 (3.4)	0.635
Severe pneumonia, n (%)	60 (26.8)	603 (15.4)	< 0.001
**Targeted pharmacotherapy**			
Prostacyclin, n (%)	3 (1.3)	63 (1.6)	0.754
PDE-5i, n(%)	15 (6.7)	491 (12.5)	0.009
Endothelin receptor antagonists, n (%)	3 (1.3)	25 (0.6)	0.213
Combination therapy, n (%)	0 (0)	2 (0.1)	0.735
Congenital heart surgery, n (%)	5 (2.2)	481 (12.3)	< 0.001
Mechanical ventilation, n (%)	13 (5.8)	1044 (26.7)	< 0.001
Nonmedical order discharge, n (%)	55 (24.6)	1277 (32.6)	0.012
LOS (days), median [Q1, Q3]	10 [7, 13]	12 [7, 20]	0.001

Abbreviations: n, number; Q1, the first quartile; Q3, the third quartile; IPAH, idiopathic pulmonary arterial hypertension; CHD: congenital heart disease; BPD: Bronchopulmonary Dysplasia; PDE-5i: phosphodiesterase 5 inhibitors; LOS: length of stay.

Supplementary Table 2 Baseline characteristic in validation set.

**Variables**	**Readmission (n = 96)**	**Non-readmission (n = 1678)**	**P-value**
Age (years), median [Q1, Q3]	0.35 [0.12, 0.65]	0.12 [0.00, 0.62]	< 0.001
Male, n (%)	56 (58.3)	889 (53.0)	0.307
IPAH, n (%)	0 (0)	24 (1.4)	0.238
Connective tissue disease, n (%)	0 (0)	1 (0.1)	0.811
Dilated cardiomyopathy, n (%)	0 (0)	5 (0.3)	0.592
CHD, n (%)	95 (99.0)	1642 (97.9)	0.462
BPD, n (%)	6 (6.3)	39 (2.3)	0.017
Interstitial lung disease, n (%)	2 (2.1)	8 (0.5)	0.041
Obstructive sleep apneas, n (%)	0 (0)	1 (0.1)	0.811
Asthma, n (%)	3 (3.1)	11 (0.7)	0.008
Hypothyroidism, n (%)	0 (0)	6 (0.4)	0.557
Persistent PH in newborn, n (%)	0 (0)	11 (0.7)	0.426
Congenital diaphragmatic hernia, n (%)	1 (1.0)	8 (0.5)	0.449
Chromosomal abnormalities, n (%)	2 (2.1)	100 (6.0)	0.113
Preterm birth, n (%)	6 (6.3)	244 (14.5)	0.023
Low-weight-birth infants, n (%)	3 (3.1)	128 (7.6)	0.101
Very-low-birth-weight infants, n (%)	0 (0)	28 (1.7)	0.202
Sepsis, n (%)	6 (6.3)	307 (18.3)	0.003
Intracranial hemorrhage, n (%)	6 (6.3)	375 (22.3)	< 0.001

Arrhythmia, n (%)	0 (0)	25 (1.5)	0.228
Multi-organ dysfunction syndromes, n (%)	0 (0)	2 (0.1)	0.735
Respiratory failure, n (%)	22 (22.9)	616 (36.7)	0.006
Heart failure, n (%)	4 (4.2)	44 (2.6)	0.364
Severe pneumonia, n (%)	21 (21.9)	242 (14.4)	0.046
**Targeted pharmacotherapy**			
Prostacyclin, n (%)	0 (0)	27 (1.6)	0.210
PDE-5i, n(%)	6 (6.3)	225 (13.4)	0.043
Endothelin receptor antagonists, n (%)	1 (1.0)	6 (0.4)	0.298
Combination therapy, n (%)	0 (0)	1 (0.1)	0.811
Congenital heart surgery, n (%)	3 (3.1)	208 (12.4)	0.006
Mechanical ventilation, n (%)	6 (6.3)	465 (27.7)	< 0.001
Nonmedical order discharge, n (%)	16 (16.7)	528 (31.5)	0.002
LOS (days), median [Q1, Q3]	9 [7, 13]	12 [7, 21]	< 0.001

Abbreviations: n, number; Q1, the first quartile; Q3, the third quartile; IPAH, idiopathic pulmonary arterial hypertension; CHD, congenital heart disease; BPD, bronchopulmonary dysplasia; PDE-5i, phosphodiesterase 5 inhibitors; LOS, length of stay.

Supplementary Table 3 Baseline characteristic between the training set and validation set.

Variables	Training set (n = 4139)	Validation set (n = 1774)	P-value
Age (years), median [Q1, Q3]	0.10 [0, 0.58]	0.13 [0.00, 0.62]	0.043
Male, n (%)	2310 (55.9)	945 (53.3)	0.076
IPAH, n (%)	33 (0.8)	24 (1.4)	0.063
Connective tissue disease, n (%)	4 (0.1)	1 (0.1)	1
Dilated cardiomyopathy, n (%)	4 (0.1)	5 (0.3)	0.139
CHD, n (%)	4043 (97.7)	1737 (97.9)	0.646
BPD, n (%)	92 (0.2)	45 (2.5)	0.522
Interstitial lung disease, n (%)	24 (0.6)	10 (0.6)	1
Obstructive sleep apneas, n (%)	4 (0.1)	1 (0.1)	1
Asthma, n (%)	21 (0.5)	14 (0.8)	0.267
Hypothyroidism, n (%)	19 (0.5)	6 (0.3)	0.662
Persistent PH in newborn, n (%)	41 (0.9)	11 (1.0)	0.213
Congenital diaphragmatic hernia, n (%)	19 (0.5)	9 (0.5)	0.967
Chromonosomal abnormalities, n (%)	247 (6.0)	102 (6.0)	0.791
Preterm birth, n (%)	686 (16.6)	250 (14.1)	0.018
Low-weight-birth infants, n (%)	342 (8.3)	131 (7.4)	0.276
Very-low-birth-weight infants, n (%)	71 (1.7)	28 (1.6)	0.790

Sepsis, n (%)	748 (18.1)	313 (17.6)	0.722
Intracranial hemorrhage, n (%)	987 (23.8)	381 (21.5)	0.052
Arrhythmia, n (%)	43 (1.0)	25 (1.4)	0.275
Multi-organ dysfunction syndromes, n (%)	5 (0.1)	2 (0.1)	1
Respiratory failure, n (%)	1503 (36.3)	638 (36.0)	0.821
Heart failure, n (%)	143 (3.5)	48 (2.7)	0.158
Severe pneumonia, n (%)	663 (16.0)	263 (14.8)	0.264
**Targeted pharmacotherapy**			
Prostacyclin, n (%)	66 (1.6)	27 (1.5)	0.927
PDE-5i, n(%)	506 (12.2)	231 (13.0)	0.420
Endothelin receptor antagonists, n (%)	28 (0.7)	7 (0.4)	0.267
Combination therapy, n (%)	2 (0.04)	1 (0.1)	1
Congenital heart surgery, n (%)	486 (11.7)	211 (11.9)	0.903
Mechanical ventilation, n (%)	1057 (25.5)	471 (26.6)	0.434
Nonmedical order discharge, n (%)	1332 (32.2)	544 (30.7)	0.264
LOS (days), median [Q1, Q3]	12 [7, 20]	12 [7, 21]	0.287

Abbreviations: n, number; Q1, the first quartile; Q3, the third quartile; IPAH, idiopathic pulmonary arterial hypertension; CHD: congenital heart disease; BPD: Bronchopulmonary Dysplasia; PDE-5i: phosphodiesterase 5 inhibitors; LOS: length of stay.

Supplementary Table 4 Hyperparameter search domains and final settings.

Hyperparameters	Search domain	Final setting
'iterations'	(100, 1000)	149
'learning_rate'	(0.01, 0.1)	0.04
'depth'	(2, 15)	6
'l2_leaf_reg'	(1, 10)	7
